# Supplementary material for: SPIDR: a highly multiplexed method for mapping RNA-protein interactions uncovers a potential mechanism for selective translational suppression upon cellular stress
Source: bioRxiv. 2023 Jun 7:2023.06.05.543769. Preprint. [Version 1] doi: 10.1101/2023.06.05.543769 (PMC10274648; doi:10.1101/2023.06.05.543769)
Supplement: Supplement 1 [file NIHPP2023.06.05.543769v1-supplement-1.pdf]

bioRxiv preprint doi: https://doi.org/10.1101/2023.06.05.543769; this version posted June 7, 2023. The copyright holder for this preprint (which was not certified by peer review) is the author/funder, who has granted bioRxiv a license to display the preprint in perpetuity. It is made available under aCC-BY 4.0 International license.

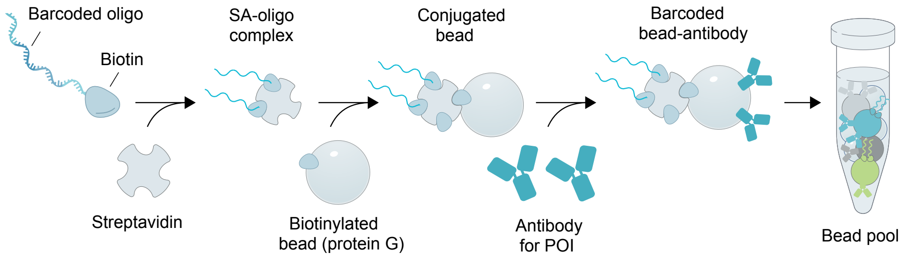

Supplemental Figure 1: Schematic of our multiplexed antibody-bead labeling strategy.
Populations of biotinylated protein G beads are incubated with a streptavidin-biotin oligo complex. Each population of beads is labeled with an oligo with a specific sequence and then incubated with one type of capture antibody such that each population has a unique capture antibody and a corresponding oligo tag that can be recognized after sequencing. Populations are combined to create the bead pool.

bioRxiv preprint doi: https://doi.org/10.1101/2023.06.05.543769; this version posted June 7, 2023. The copyright holder for this preprint (which was not certified by peer review) is the author/funder, who has granted bioRxiv a license to display the preprint in perpetuity. It is made available under aCC-BY 4.0 International license.

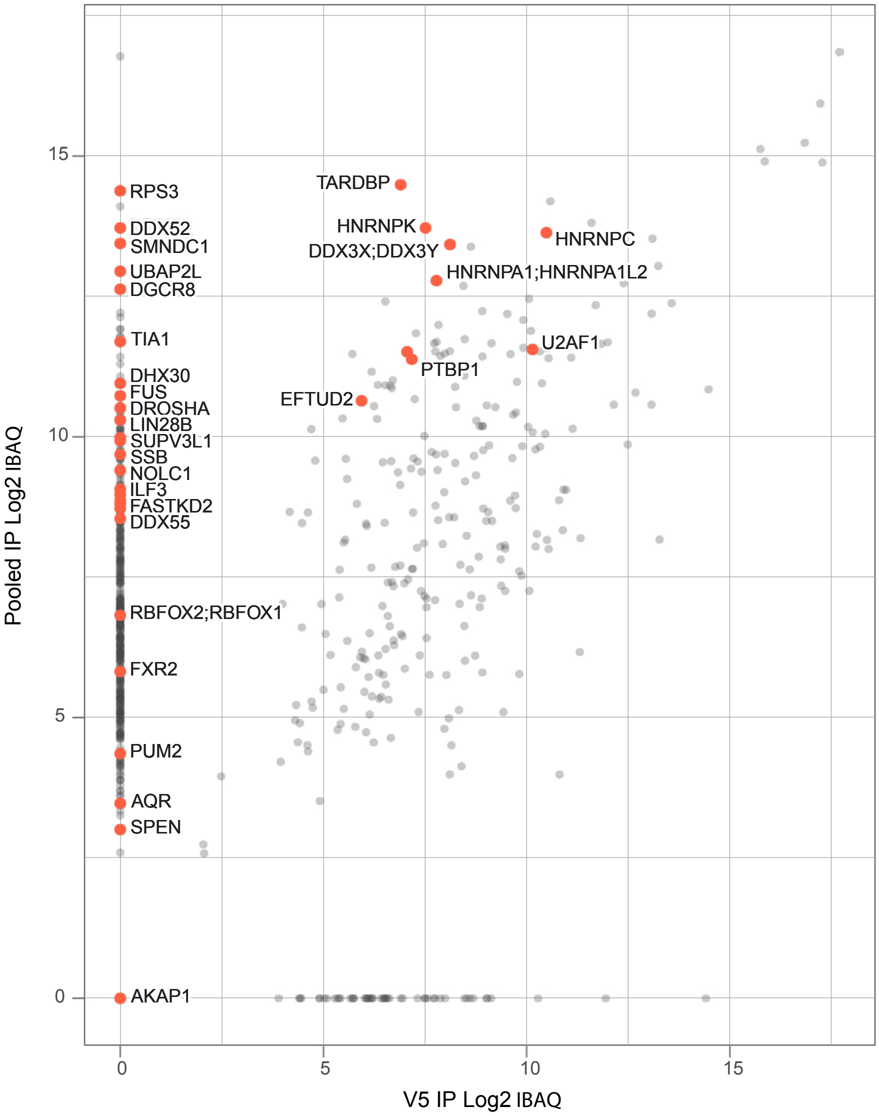

Supplemental Figure 2: Multiplexed IP of dozens of RBPs accurately recovers targeted proteins.
Scatter plot showing log2 transformed IBAQ (intensity based absolute quantification)[82] values for all identified proteins in either the pooled IP with 39 targets (y-axis) versus those detected with a V5 negative control IP (x-axis) by LC-MS/MS. Target proteins that should be detected by the antibodies included in the pool of 39 used are marked in red.

bioRxiv preprint doi: https://doi.org/10.1101/2023.06.05.543769; this version posted June 7, 2023. The copyright holder for this preprint (which was not certified by peer review) is the author/funder, who has granted bioRxiv a license to display the preprint in perpetuity. It is made available under aCC-BY 4.0 International license.

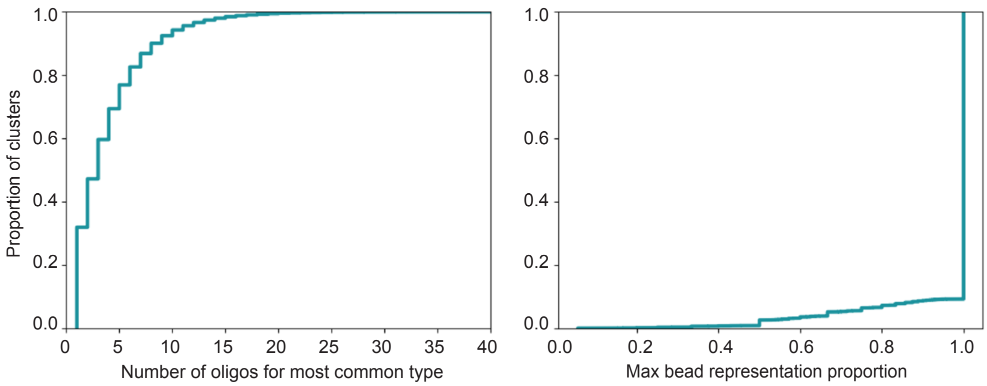

Supplemental Figure 3: Uniqueness of beads and number of oligos per bead of the experiment.
Observed distributions of labeled beads after sequencing. Each bead is defined in sequencing by a particular, unique combinatorial barcode acquired during split-pool. A SPIDR cluster represents any set of molecules, oligo or RNA, that share the same bead combinatorial barcode. Left: CDF plot showing the number of independent oligos matched within an individual SPIDR cluster. Right: CDF plot describing the degree of heterogeneity of these detected oligos within each SPIDR cluster, as determined by oligos with a shared combinatorial barcode. X axis represents the homogeneity of the oligo types with 1 indicating that all oligos are of the same type.

bioRxiv preprint doi: https://doi.org/10.1101/2023.06.05.543769; this version posted June 7, 2023. The copyright holder for this preprint (which was not certified by peer review) is the author/funder, who has granted bioRxiv a license to display the preprint in perpetuity. It is made available under aCC-BY 4.0 International license.

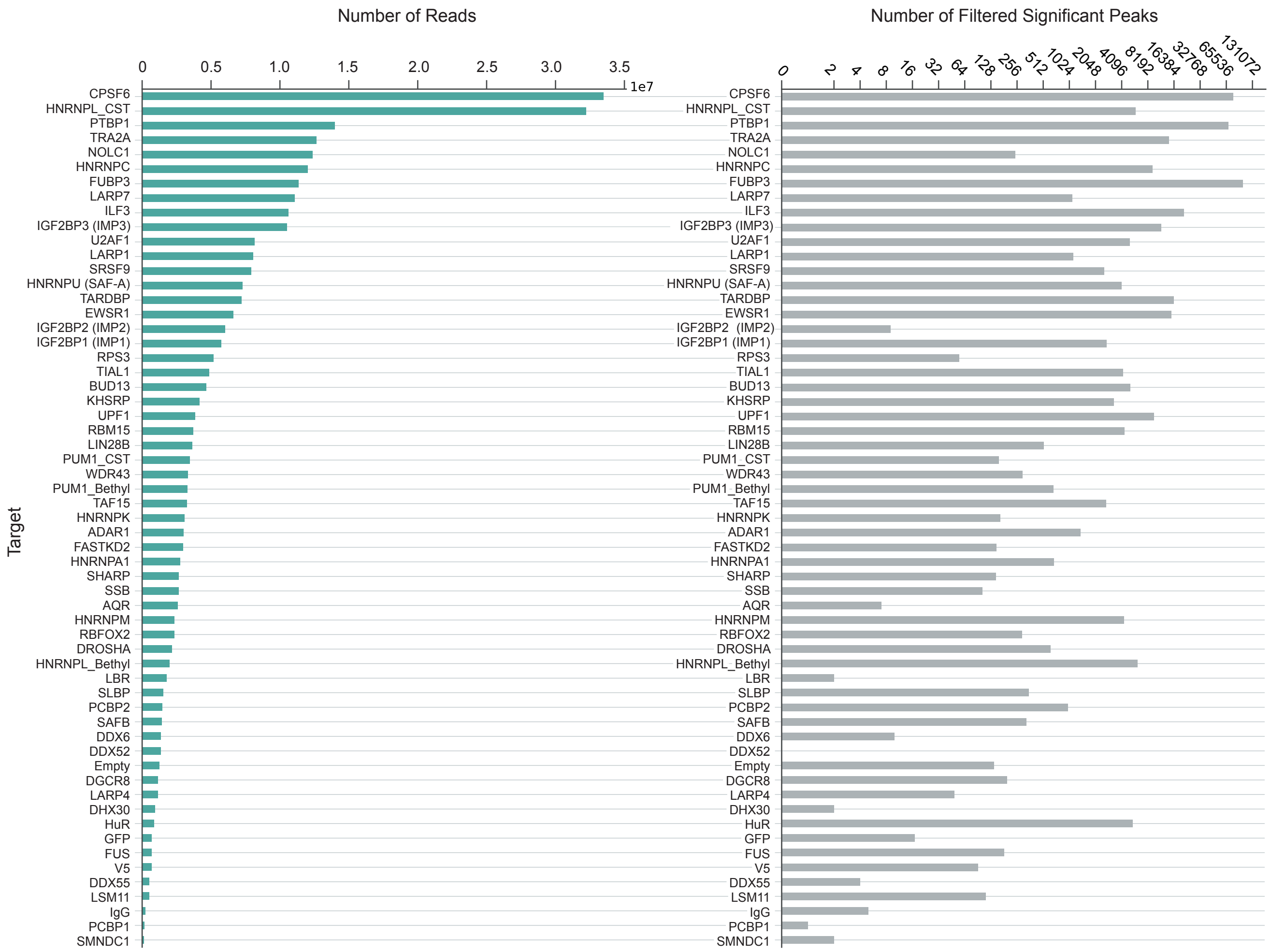

Supplemental Figure 4: Mapped unique reads per RBP and significant binding sites identified per RBP.
Number of deduplicated mapped reads and number of significant binding sites within uniquely mapped genomic regions per IP. The order is determined by the number of unique mapped reads in both plots.

bioRxiv preprint doi: https://doi.org/10.1101/2023.06.05.543769; this version posted June 7, 2023. The copyright holder for this preprint (which was not certified by peer review) is the author/funder, who has granted bioRxiv a license to display the preprint in perpetuity. It is made available under aCC-BY 4.0 International license.

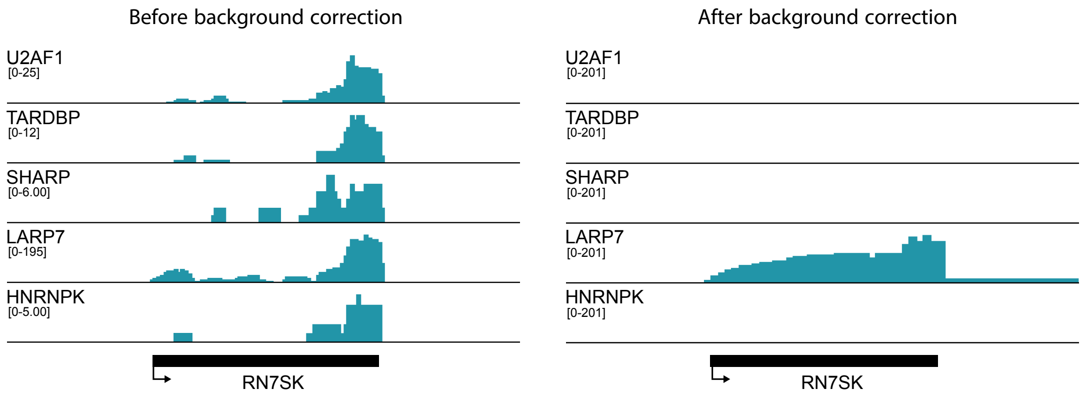

Supplemental Figure 5: Background correction.
An example of our background correction method that utilizes the total read coverage across all proteins to normalize each individual protein. Shown are example tracks on RN7SK before and after background correction. Left: Raw alignment data for the entire pooled dataset (top track) and for representative antibodies against U2AF1, TARDBP, SHARP, LARP7 and HNRNPK on *RN7SK*. Right: Background corrected data for the same set of antibodies. Signal that was not antibody-specific has been normalized out. The reads in the right are binned in 5 nucleotide windows. *RN7SK* is known to be bound by LARP7[Ref.51].

bioRxiv preprint doi: https://doi.org/10.1101/2023.06.05.543769; this version posted June 7, 2023. The copyright holder for this preprint (which was not certified by peer review) is the author/funder, who has granted bioRxiv a license to display the preprint in perpetuity. It is made available under aCC-BY 4.0 International license.

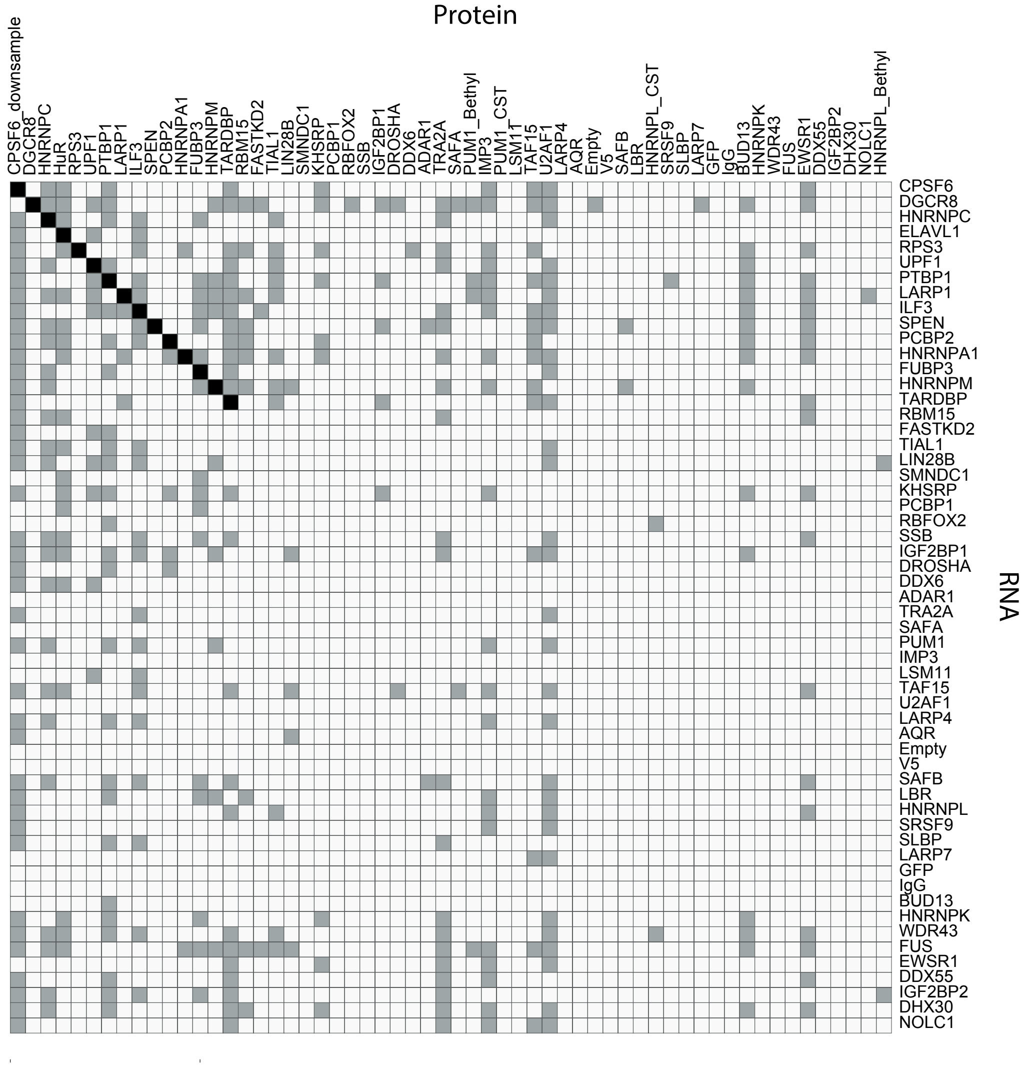

Supplemental Figure 6: Autoregulatory binding between RBPs targeted by SPIDR and their RNAs.
Auto-regulatory binding matrix with protein (x-axis) binding to each mRNA (y-axis) shown. Each target protein included in SPIDR performed in K562 cells marked by whether it has significantly enriched binding within its own RNA, or in any of the other SPIDR target RNAs. Proteins that bind their own RNA are marked in black, instances of binding to genes of other SPIDR targets are marked in gray.

bioRxiv preprint doi: https://doi.org/10.1101/2023.06.05.543769; this version posted June 7, 2023. The copyright holder for this preprint (which was not certified by peer review) is the author/funder, who has granted bioRxiv a license to display the preprint in perpetuity. It is made available under aCC-BY 4.0 International license.

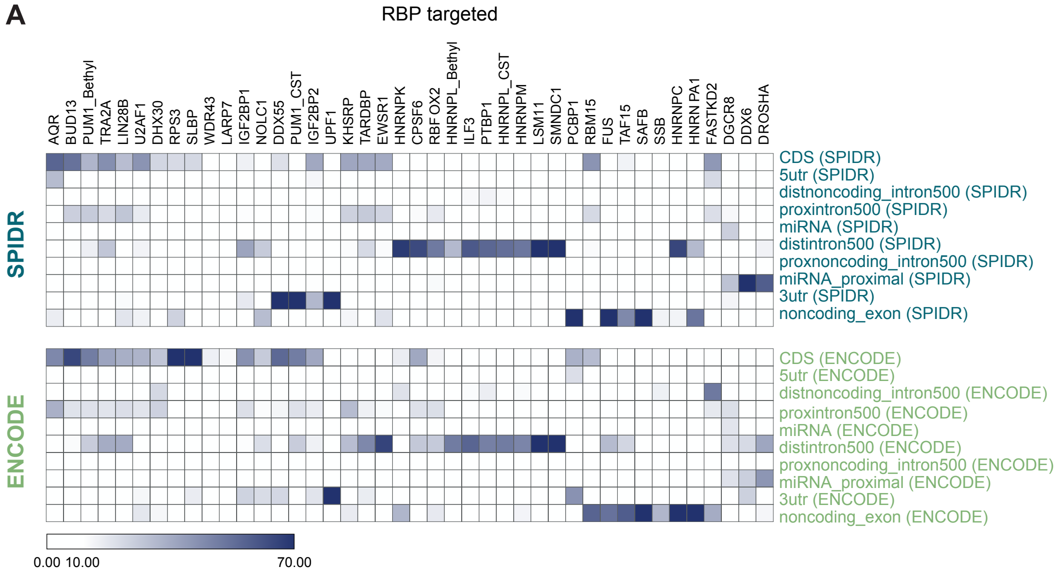

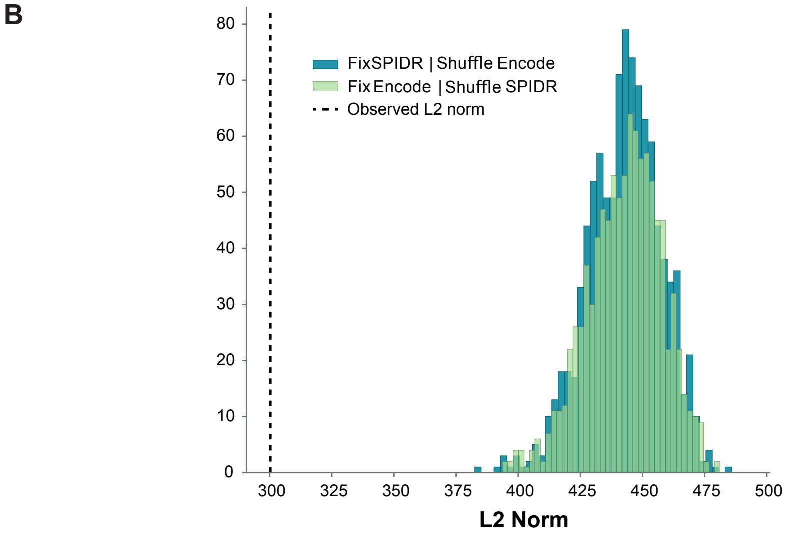

Supplemental Figure 7: Global comparison of annotations (intron, exon, etc) of binding sites per RBP as called by ENCODE versus SPIDR

(A) Heatmaps showing the percentage of significant binding sites in each of the annotation categories for SPIDR performed in K562 cells and ENCODE (see **Methods** for details). **(B**) Quantitative assessment of the similarity of heatmaps between SPIDR and ENCODE. The Euclidean distance (L2 norm) between the ENCODE and SPIDR percentage tables/heatmaps was calculated. The calculated distance is indicated by the dashed line. The statistical significance was calculated by randomly shuffling the columns of either the SPIDR percentage table and keeping the original ENCODE table or vice versa, meaning shuffling the columns of the ENCODE table and keeping the original SPIDR table. This was done 1000 times in each direction and every time the Euclidean distance was calculated. The values are represented by the two histograms. The Euclidean distance of all of the randomly shuffled 2000 comparison was always larger than of the true pair, which shows that the two original annotation tables from SPIDR and ENCODE are highly significantly similar (p-value < 0.0005).

bioRxiv preprint doi: https://doi.org/10.1101/2023.06.05.543769; this version posted June 7, 2023. The copyright holder for this preprint (which was not certified by peer review) is the author/funder, who has granted bioRxiv a license to display the preprint in perpetuity. It is made available under aCC-BY 4.0 International license.

Supplemental Table 1:
Overview of the SPIDR experiment in K562 cells. The protein targets are listed, as well as the vendors and product numbers of the corresponding antibodies.

Target RBP	Antibody Vendor	Antibody Catalog Number
HNRNPM	Santa Cruz	sc-20001
HNRNPC	Abcam	ab10294
HNRNPL_Bethyl	Bethyl	A303-896A
RBM15	Bethyl	A300-821A
DHX30	Bethyl	A302-218A
SAFB	Invitrogen	MA1-91526
PCBP1	Abcam	168378
V5	Invitrogen	MA5-15253
SRSF9	Abcam	ab74782
ADAR1	CST	D7E2M
HuR	CST	D9W7E
AQR	Bethyl	A302-547A
FUBP3	Bethyl	A303-012A
ILF3	Bethyl	A303-651A
LARP4	Bethyl	A303-723A
BUD13	Bethyl	A303-320A
DDX52	Bethyl	A303-054A
DDX55	Bethyl	A303-027A
DDX6	Bethyl	A300-460A
DGCR8	Bethyl	A302-468A
DROSHA	Bethyl	A301-886A
EWSR1	Bethyl	A300-417A
FASTKD2	Bethyl	A303-788A
LARP7	Bethyl	A303-723A
TRA2A	Bethyl	A303-779A
U2AF1	Bethyl	A302-079A
UPF1	Bethyl	A301-902A
LIN28B	Bethyl	A303-588A
NOLC1	Bethyl	A302-184A
PUM1_Bethyl	Bethyl	A302-576A
RBFOX2	Bethyl	A300-864A
RPS3	Bethyl	A303-840A
SHARP	Bethyl	A301-119A
TAF15	Bethyl	A300-308A
TARDBP	Bethyl	A303-223A
CPSF6	CST	92879S
IGF2BP1 (IMP1)	MBL International	RN007P
KHSRP	Abcam	ab150393
LARP1	Bethyl	A303-900A
FUS	CST	67840S
HNRNPL_CST	CST	65043S
IMP3	CST	57145S
PTBP1	CST	57246S
PUM1_CST	CST	12322S
TIAL1	CST	8509S
HNRNPA1	Abcam	ab4791
HNRNPK	MBL International	RNP019
PCBP2	MBL International	RN025P
WDR43	Bethyl	A302-478A
LSM11	Bethyl	A303-709A
Empty	n/a	n/a
SSB	MBL International	RN074PW
SLBP	MBL International	RN045P
SMNDC1	MBL International	RN078PW
GFP	Living Colors	JL-8
IgG	Abcam	ab172730
IGF2BP1 (IMP2)	MBL International	RN008P
LBR	Abcam	ab122919
HNRNPU (SAF-A)	Abcam	ab20666

Supplemental Table 2:
Overview of the SPIDR experiment HEK293T cells treated with Torin or Control (solvent only). The protein targets are listed, as well as the vendors and product numbers of the corresponding antibodies.

Target RBP	Antibody Vendor	Antibody Catalog Number
AQR	Bethyl	A302-547A
SAF-A	Abcam	ab20666
HNRNPM3/4	Santa Cruz	sc-20001
BUD13	Bethyl	A303-320A
DDX52	Bethyl	A303-054A
DDX55	Bethyl	A303-027A
DDX6	Bethyl	A300-460A
DGCR8	Bethyl	A302-468A
DROSHA	Bethyl	A301-886A
EWSR1	Bethyl	A300-417A
V5	Invitrogen	MA5-15253
FASTKD2	Bethyl	A303-788A
SHARP	Bethyl	A301-119A
TAF15	Bethyl	A300-308A
FUBP3	Bethyl	A303-900A
ILF3	Bethyl	A303-651A
LARP4	Bethyl	A303-900A
LARP7	Bethyl	A303-723A
LIN28B	Bethyl	A303-588A
NOLC1	Bethyl	A302-184A
RBFOX2	Bethyl	A300-864A
RPS3	Bethyl	A303-840A
TRA2A	Bethyl	A303-779A
HNRNPL	Bethyl	A303-896A
IMP3	CST	57145S
PTBP1	CST	57246S
U2AF1	Bethyl	A302-079A
UPF1	Bethyl	A301-902A
EIF4G1	CST	2858S
RPS2	Bethyl	A303-794A
RBM15	Bethyl	A300-821A
CPSF6	CST	92879S
HNRNPC	Abcam	ab10294
PUM1	Bethyl	A302-576A
SLBP	MBL International	RN045P
SMNDC1	MBL International	RN078PW
GFP	Living Colors	JL-8
TIAL-1	CST	8509S
HNRNPK	MBL International	RNP019
IMP1	MBL International	RN007P
KHSRP	Abcam	ab150393
LARP1	Bethyl	A303-900A
SSB	MBL International	RN074PW
IgG	Abcam	ab172730
XRN1	CST	70205S
RPS6	CST	2217S
Empty	n/a	n/a
IMP2	MBL International	RN008P
SAFB	Invitrogen	MA1-91526
LBR	Abcam	ab122919
PCBP1	Abcam	168378
4EBP1	CST	9644S
EIF4E	CST	2067S
EIF4A	CST	2013S
